# SERUM 25(OH)D SIGNIFICANTLY IMPACTS ALZHEIMER’S DISEASE IN OLDER ADULTS LIVING IN LONG-TERM CARE COMMUNITIES.

**DOI:** 10.1093/geroni/igz038.3170

**Published:** 2019-11-08

**Authors:** Robert A Churchill, Ronna Robbins, Nalini Ranjit, Sara Sweitzer, Margaret Briley

**Affiliations:** 1 The University of Texas at Austin, Austin, Texas, United States; 3 The University of Texas Health Science Center at Houston School of Public Health in Austin, Austin, Texas, United States

## Abstract

6.08 million Americans suffer from Alzheimer’s Disease (AD), with some estimating diagnoses will reach 15.0 million by 2060. Age is the strongest risk factor for AD, and the prevalence of AD among older adults necessitates investigation into preventable risk factors. 25-hydroxyvitamin D [25(OH)D] deficiency is more prevalent in older adults than other age demographics. Research shows a strong correlation between deficient 25(OH)D serum levels (≤20ng/ml) and AD diagnosis. The association with insufficient (≤30 ng/ml) levels remains unclear. Older adults (age > 65 yo) of five LTC communities in Texas participated in the cross-sectional study. One-year medical history and demographics were abstracted from medical records using double-blinded data abstraction and entry. Blood draws measured 25(OH)D serum levels. Adjusted logistic regression models examined if insufficient 25(OH)D serum levels (≤30 ng/ml) are associated with AD diagnosis. Confounders were total daily vitamin D supplementation, BMI, race, gender, age, years in community, and diagnosis of liver and renal disease. Participants (n=174, mean age: 83 yo) consisted of 63% female and 89% Caucasian. Fifty five percent had insufficient serum 25(OH)D levels (mean level: 32.6 ng/ml; mean supplementation rate: 1,138 IU per/d), and 20% had diagnosis of AD. 25% had both insufficient serum levels and AD, while 12.6% had adequate serum levels and AD. Those with insufficient 25(OH)D serum levels had elevated odds (OR=2.8; CL: 1.14, 7.02; p=0.024) of having AD after adjusting for confounders. Insufficient serum 25(OH)D levels (≤30 ng/ml) are associated with increased diagnoses of AD, indicating the importance of adequate levels among LTC residents.

